# Supplementary material for: eIF5A controls mitoprotein import by relieving ribosome stalling at the TIM50 translocase mRNA
Source: bioRxiv. 2023 Dec 19:2023.12.19.572290. Preprint. [Version 1] doi: 10.1101/2023.12.19.572290 (PMC10769225; doi:10.1101/2023.12.19.572290)
Supplement: Supplement 1 [file media-1.pdf]

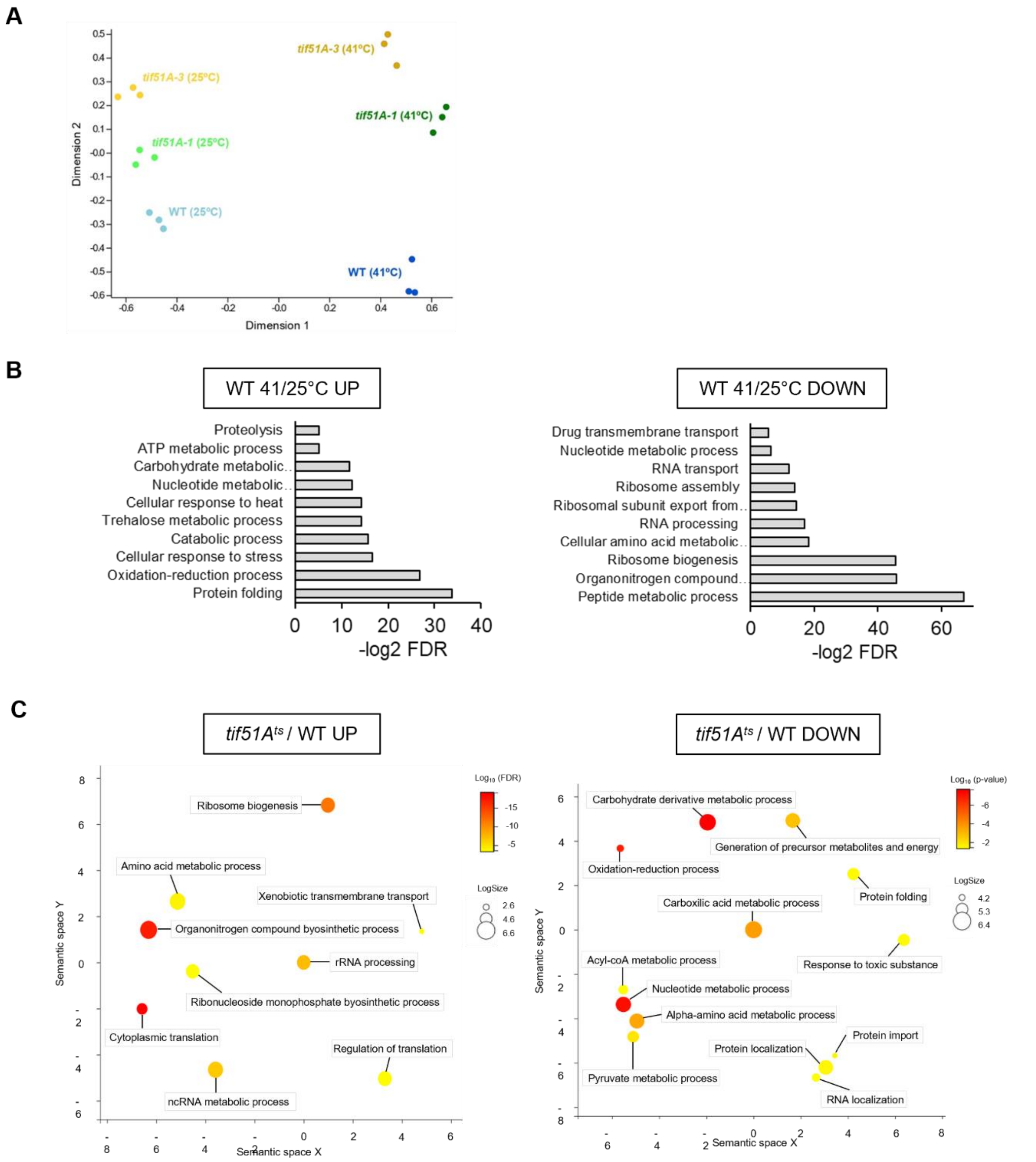

Figure EV1. Proteomic analysis upon eIF5A depletion.

(A) MDS-plot showing all replicates for each strain and condition studied in the proteomic analysis.

(B) Biological Process overrepresented in proteins up- or down- regulated significantly in wild-type cells at 41°C. GO Term Analysis was done using the STRING tool, and a total of 272 proteins up- (left) and 154 proteins down-regulated (right) significantly in WT 41°C compared to WT 25°C were analysed.

(C) Biological process Gene Ontology (GO) Terms overrepresented in relative up- (left) or down-regulated (right) proteins in *tif51A*^{ts} vs WT. GO Term analysis was done using the STRING tool, in which a total of 292 proteins down- and 135 proteins up- regulated significantly in at least one *tif51A*^{ts} with respect to WT were analysed. The web based tool REVIGO was used to summarize the GO terms. Bubble colour indicates the p-value of the GO term in the input data set; bubble size indicates the frequency of the GO term in the underlying GO database.

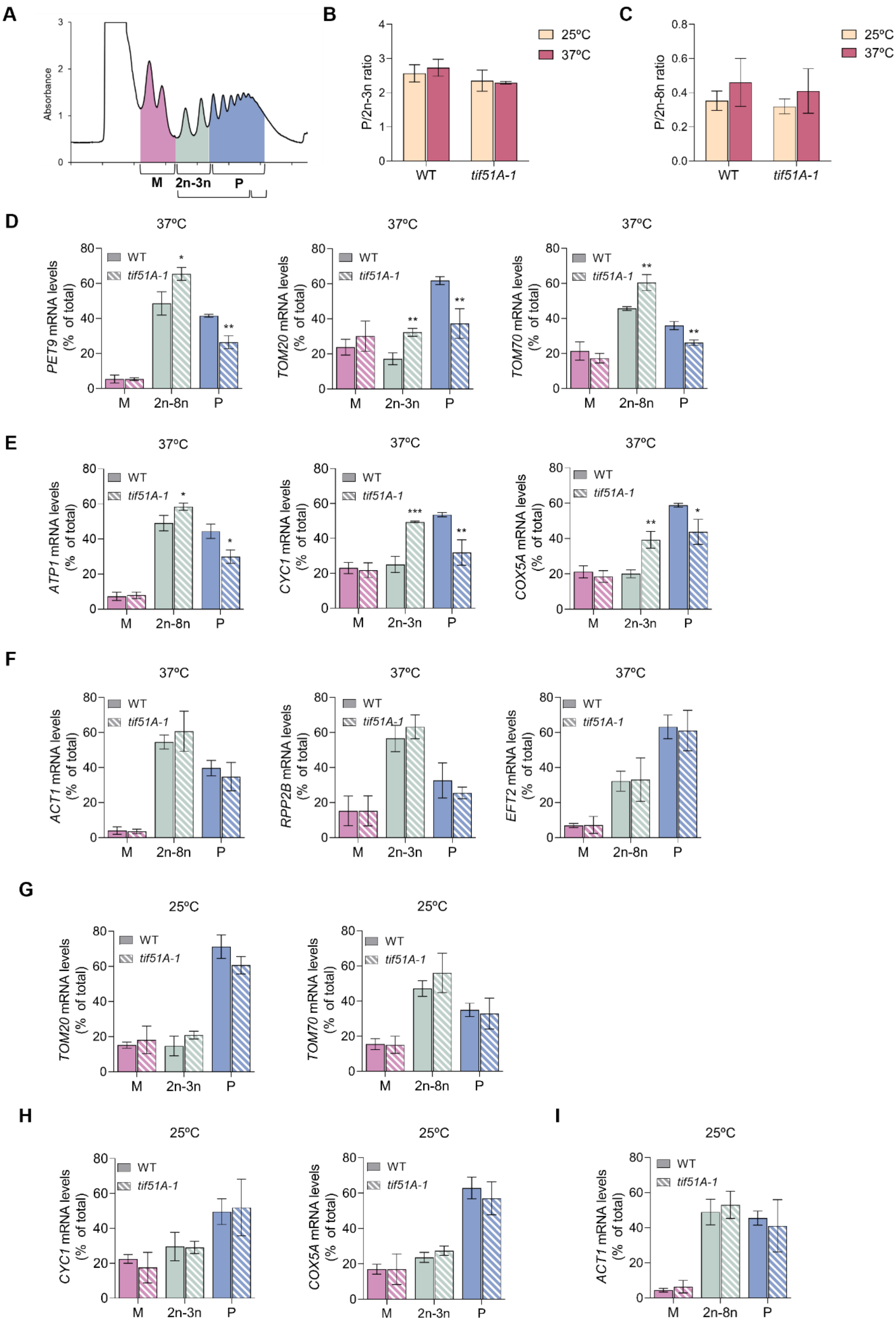

Figure EV2. Translation of mitochondrial proteins is not affected in the *tif51A-1* strain at permissive temperature.

(A) Scheme with the employed area divisions for calculations.

(B) The average polysomes/2n-3n ratio (P/2n-3n) is represented for each strain at both temperatures.

(C) The average polysomes/2n-8n ratio (P/2n-3n) is represented for each strain at both temperatures.

(D-F) The RNA from individual fractions of the polysomes profiles was extracted and the mRNA levels of *PET9*, *TOM20, TOM70* (D), *ATP1, CYC1, COX5A* (E), *ACT1, RPP2B* and *EFT2* (F) were analyzed by RT-qPCR in the corresponding sections at restrictive temperature. (G-I) The RNA from individual fractions of the polyribosome profiles was extracted and the mRNA levels of *TOM20*, *TOM70* (G), *CYC1*, COX5A (H) and *ACT1* (I) were analyzed by RT-qPCR in the corresponding sections at permissive temperature.

Data information: In (B-I) Results are presented as mean ± SD from three independent experiments. The statistical significance was measured by using a two-tailed paired Student t-test relative to wild-type strain. *$p < 0.05$, **$p < 0.001$, ***$p < 0.001$.

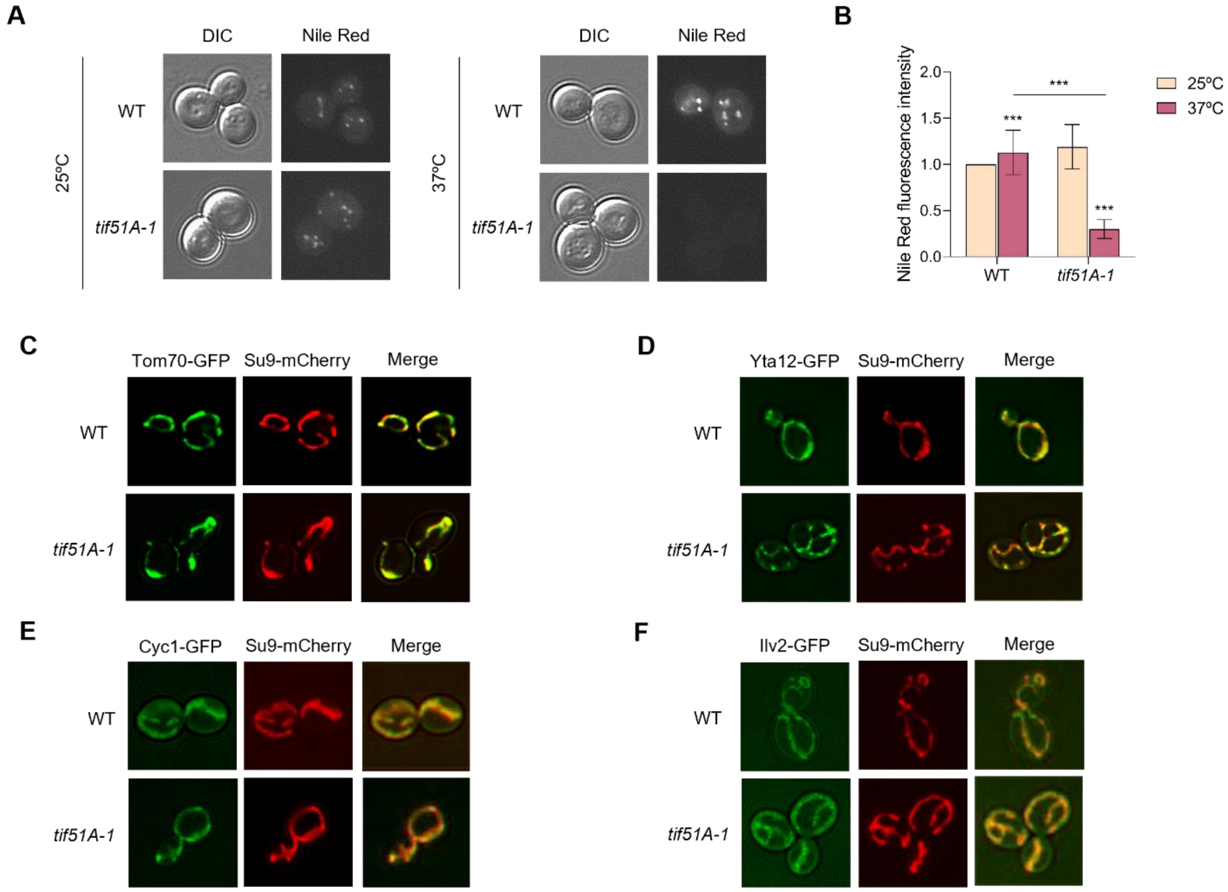

Figure EV3. Nile Red is excluded from the *tif51A-1* mutant because of high Pdr5 pumping activity.

(A) Wild-type strain and *tif51A-1* were cultured in SGal medium at 25°C until reaching post-diauxic phase and then transferred to 25°C or 37°C for 4 h. Then, cells were incubated with Nile Red substrate for 15 min prior to microscopy. A representative image is shown.

(B) Quantification of Nile Red fluorescent signal from at least 150 cells.

(C-F) Wild-type strain and *tif51A-1* expressing Tom70-GFP (C), Yta12-GFP (D), Cyc1-GFP (E) or Ilv2-GFP (F) and Su9-mCherry were cultured in SGal medium at 25°C until reaching post-diauxic phase and subjected to fluorescence microscopy.

Data information: In (B) Results are presented as mean ± SD from three independent experiments. The statistical significance was measured by using a two-tailed paired Student t-test relative to 25°C. ***$p < 0.001$.

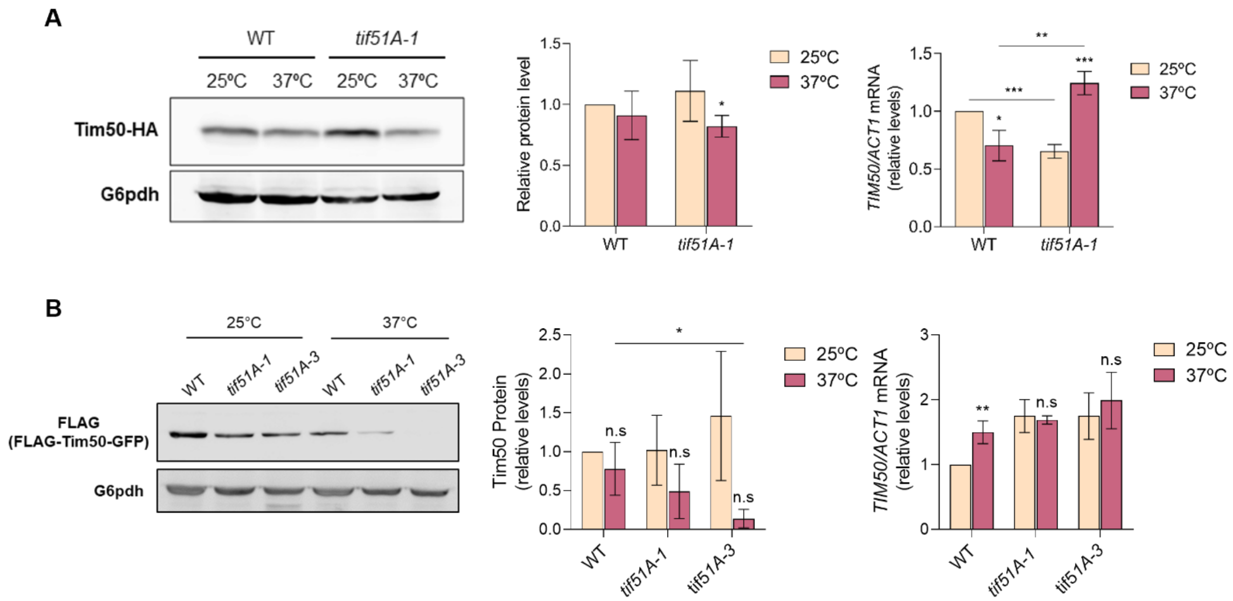

Figure EV4. Tim50 protein levels are reduced upon eIF5A depletion.

(A) Wild-type and *tif51A-1* strains containing genomic tagged Tim50-HA were cultured in SGal until post-diauxic phase at 25°C and transferred to 25°C or 37°C for 4 h. Tim50 protein levels were determined by western blotting (left) and quantified (middle). G6PDH levels were used as loading control. A representative image is shown. *TIM50* mRNA relative levels were determined by RT-qPCR (right).

(B) Wild-type, *tif51A-1* and *tif51A-3* strains harbouring a FLAG-TIM50-GFP plasmid were cultured in SRaf-URA at 25ºC until early exponential phase, transferred to 25°C and 37°C for 2 h and then transferred to SGal-URA at 25°C and 37°C for 3 additional hours. Tim50 protein levels were determined by western blotting using a FLAG antibody (left) and quantified (middle). G6PDH levels were used as loading control. A representative image is shown. *TIM50* mRNA relative levels were determined by RT-qPCR (right).

Data information: In (A,B) Results are presented as mean ± SD from three independent experiments. The statistical significance was measured by using a two-tailed paired Student t-test relative to 25ºC. *p < 0.05, **p < 0.01, ***p < 0.001.

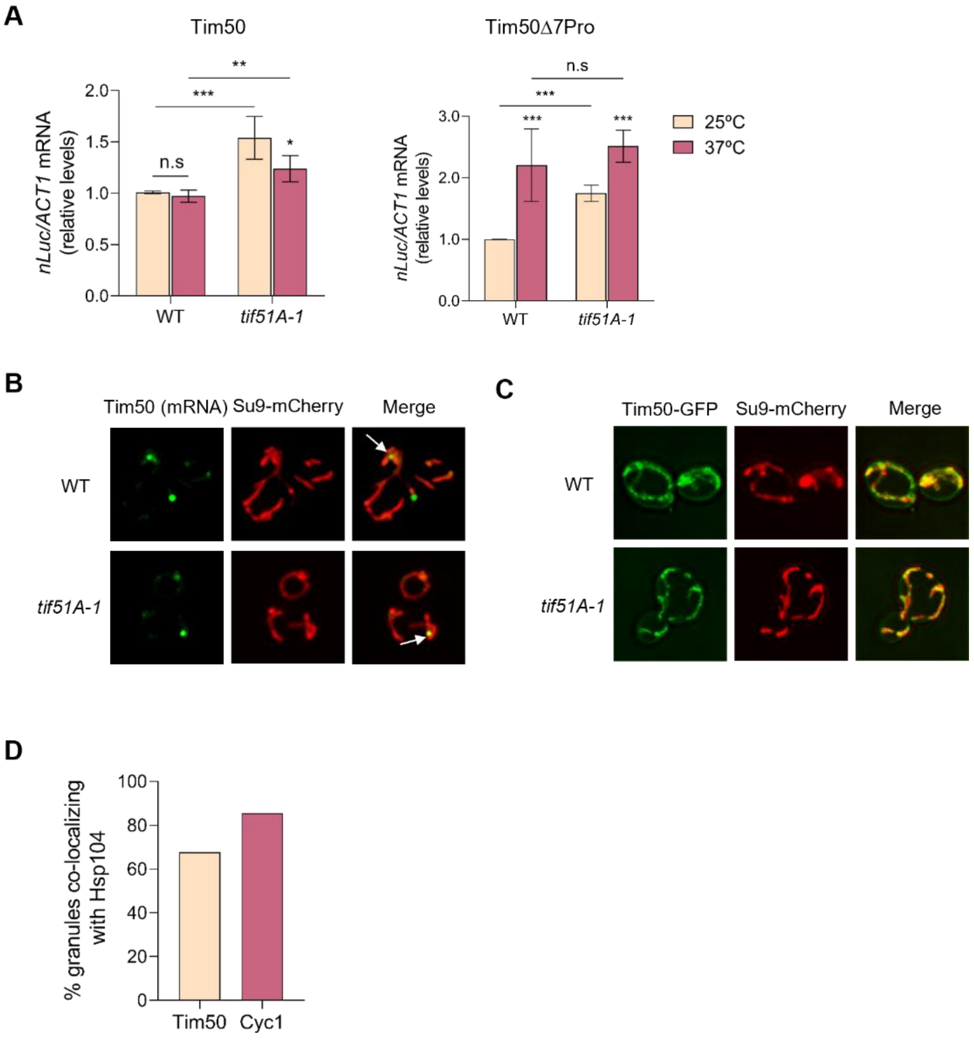

Figure EV5. eIF5A depletion does not affect *TIM50* mRNA levels nor its mitochondrial mRNA localization.

(A) Wild-type strain and *tif51A-1* expressing the wild-type Tim50 (left) or Tim50Δ7Pro (right) nLuc constructs were cultured in YPD at 25°C or 37°C for 4 h. One hour after addition of doxycycline to induce nanoluciferase expression, nLuc mRNA relative levels were determined by RT-qPCR.

(B) Wild-type strain and *tif51A-1* were cultured in SGal medium until reaching post-diauxic phase at 25°C, transferred to 37°C for 4 h and then subjected to phase contrast and fluorescence microscopy. Mitochondria were visualized by Su9-mCherry and *TIM50* mRNAs were visualized by the single molecule MS2 tag system.

(C) Wild-type strain and *tif51A-1* expressing Tim50-GFP and Su9-mCherry were cultured in SGal medium at 25°C until reaching post-diauxic phase and subjected to fluorescence microscopy.

(D) Quantification of Tim50 and Cyc1 aggregates co-localizing with Hsp104 from at least 150 cells.

Data information: In (A) Results are presented as mean ± SD from three independent experiments. The statistical significance was measured by using a two-tailed paired Student t-test relative to 25ºC. $^*p < 0.05$, $^{**}p < 0.01$, $^{***}p < 0.001$.

Table S1. Mitochondrial proteins detected in the proteomic analysis with a statistically different 41°C/25°C ratio in at least one eIF5A temperature-sensitive mutant with respect to wild-type. Proteins are listed according to their functional category. The p-value was determined by a Student's t-test.

	Gene	Strain	Relative 41/25 protein ratio vs WT [1]	p-value	Location [2]	Description [2]	Putative eIF5A motifs [3]
Transport	TIM50 *	*tif51A-1*	0.824	0.2719	MIM	Mitochondrial import inner membrane translocase	9
		tif51A-3	0.862	0.2747			
	TOM70	*tif51A-1*	0.676	0.0206	MOM	Component of the TOM (translocase of outer membrane) complex	4
		tif51A-3	0.696	0.0299			
	MIR1	*tif51A-1*	0.660	0.0022	MIM	Mitochondrial phosphate carrier; imports inorganic phosphate into mitochondria	3
		tif51A-3	0.508	0.0006			
	PET9	*tif51A-1*	0.605	0.0050	MIM	Major ADP/ATP carrier of the mitochondrial inner membrane	4
		tif51A-3	0.479	0.0010			
	POR1	*tif51A-1*	0.551	0.0017	MOM	Mitochondrial porin required for mitochondrial osmotic stability and permeability	1
		tif51A-3	0.427	0.0013			
	TOM20	*tif51A-1*	0.279	0.0011	MOM	Component of the TOM (translocase of outer membrane) complex	2
		tif51A-3	0.347	0.0012			
OXPHOS	QCR2	*tif51A-1*	0.796	0.0480	MIM	Subunit 2 of ubiquinol cytochrome-c reductase (Complex III)	1
		tif51A-3	0.703	0.0068			
	ATP1	*tif51A-1*	0.767	0.0014	MIM	Alpha subunit of the F1 sector of mitochondrial F1F0 ATP synthase	3
		tif51A-3	0.692	0.0029			
	ATP2	*tif51A-1*	0.751	0.0026	MIM	Beta subunit of the F1 sector of mitochondrial F1F0 ATP synthase	5
		tif51A-3	0.701	0.0039			
	ATP3	*tif51A-1*	0.713	0.0451	MIM	Gamma subunit of the F1 sector of mitochondrial F1F0 ATP synthase	1
		tif51A-3	0.758	0.0419			
	ATP11	*tif51A-1*	0.703	0.0057	MIM	Molecular chaperone; required for assembly of mitochondrial F1F0 ATP synthase	2
		tif51A-3	0.912	0.1657			
	COR1	*tif51A-1*	0.614	0.0005	MIM	Core subunit of the ubiquinol-cytochrome c reductase complex	0
		tif51A-3	0.473	0.0001			
	CYC1 *	*tif51A-1*	0.528	0.1694	IMS	Cytochrome c. electron carrier of mitochondrial intermembrane space	0
		tif51A-3	0.464	0.1309			
TCA	ACO1	*tif51A-1*	1.029	0.7081	MATRIX	Aconitase; also independently required for mitochondrial genome maintenance	7
		tif51A-3	0.825	0.0494			
	KGD1	*tif51A-1*	0.977	0.8031	MATRIX	Subunit of the mitochondrial alpha-ketoglutarate dehydrogenase complex	2
		tif51A-3	0.772	0.0213			
	CIT1	*tif51A-1*	0.854	0.3034	MATRIX	Mitochondrial citrate synthase; condenses acetyl-coA and oxaloacetate to form citrate	1
		tif51A-3	0.480	4.6E-05			
	IDH1	*tif51A-1*	0.571	0.0105	MATRIX	Subunit of mitochondrial NAD(+)-dependent isocitrate dehydrogenase	3
		tif51A-3	0.662	0.0241			
	LSC2	*tif51A-1*	0.534	0.0106	MATRIX	Beta subunit of succinyl-CoA ligase	4
		tif51A-3	0.299	0.0047			
Translation	THS1	*tif51A-1*	1.376	0.0345		Threonyl-tRNA synthetase; essential cytoplasmic protein	3
		tif51A-3	1.326	0.0325			
	BAT1	*tif51A-1*	1.314	0.0483	MATRIX	Mitochondrial branched-chain amino acid (BCAA) aminotransferase	2
		tif51A-3	1.256	0.0410			
	TEF4	*tif51A-1*	1.273	0.0585		Gamma subunit of translational elongation factor eEF1B	1
		tif51A-3	1.287	0.0304			
	SNL1	*tif51A-1*	1.238	0.3920		Ribosome-associated protein; proposed to act in protein synthesis	0
		tif51A-3	1.341	0.0468			
	VAS1	*tif51A-1*	1.124	0.0876		Mitochondrial and cytoplasmic valyl-tRNA synthetase	11
		tif51A-3	1.227	0.0198			
	ALA1	*tif51A-1*	1.201	0.0470		Cytoplasmic and mitochondrial alanyl-tRNA synthetase	9
		tif51A-3	1.370	0.0276			
	ILV5	*tif51A-1*	1.105	0.0649	MATRIX	Acetohydroxyacid reductoisomerase and mtDNA binding protein	2
		tif51A-3	1.267	0.0022			
	ILV2	*tif51A-1*	0.780	0.0558		Acetolactate synthase; catalyses the first step in isoleucine and valine biosynthesis	6
		tif51A-3	0.827	0.0362			

	Gene	Strain	Relative 41/25 protein ratio vs WT [1]	p-value	Location [2]	Description [2]	Putative eIF5A motifs [3]
	TMA19	*tif51A-1*	0.677	0.0769	MS	Protein associated with ribosomes; homolog of translationally controlled tumor protein	1
		tif51A-3	0.634	0.0408			
	ILV6	*tif51A-1*	0.666	0.0185	MATRIX	Acetolactate synthase. which catalyzes branched-chain amino acid biosynthesis	6
		tif51A-3	0.638	0.0135			
	TUM1	*tif51A-1*	0.651	0.0370		Rhodanese domain sulfur transferase; transfers persulfite from Nfs1p to Uba4p	0
		tif51A-3	0.810	0.1804			
	APE2	*tif51A-1*	0.548	0.0002		Aminopeptidase yscII; may have role in obtaining leucine from dipeptide substrates	6
		tif51A-3	0.534	0.0003			
	HYP2	*tif51A-1*	0.284	0.0039		Translation elongation factor eIF-5A	1
		tif51A-3	0.237	0.0029			
Chaperons	HSP78	*tif51A-1*	2.093	0.0191	MATRIX	Oligomeric mitochondrial matrix chaperone; cooperates with Ssc1p after heat shock	6
		tif51A-3	1.631	0.2857			
	HSC82	*tif51A-1*	1.342	0.0408		Cytoplasmic chaperone of the Hsp90 family; plays a role in determining prion variants	2
		tif51A-3	1.130	0.1624			
	HSP10	*tif51A-1*	0.884	0.8337	MATRIX	Mitochondrial matrix co-chaperonin; inhibits the ATPase activity of Hsp60p	0
		tif51A-3	0.496	0.0040			
	HSP60	*tif51A-1*	0.586	0.0475	MATRIX	Tetradecameric mitochondrial chaperonin	7
		tif51A-3	0.503	0.0031			
	SSC1	*tif51A-1*	0.575	0.0124	MIM	A motor component of the translocase of the Inner Mitochondrial membrane	3
		tif51A-3	0.676	0.0068			
Oxidative stress	YHB1	*tif51A-1*	1.124	0.3646	MATRIX	Nitric oxide oxidoreductase; plays role in oxidative and nitrosative stress responses	2
		tif51A-3	1.527	0.0151			
	MCR1	*tif51A-1*	0.835	0.1207	MOM; IMS	Mitochondrial NADH-cytochrome b5 reductase; involved in ergosterol biosynthesis	2
		tif51A-3	0.626	0.0032			
	SOD2	*tif51A-1*	0.824	0.3115	MATRIX	Mitochondrial manganese superoxide dismutase	3
		tif51A-3	0.446	0.0023			
	CCP1	*tif51A-1*	0.767	0.1371	IMS	Mitochondrial cytochrome-c peroxidase; degrades reactive oxygen species	3
		tif51A-3	0.533	0.0147			
	CIR2	*tif51A-1*	0.668	0.0975		Putative ortholog of human ETF-dH; may have a role in oxidative stress response	8
		tif51A-3	0.482	0.0371			
	UTH1	*tif51A-1*	0.516	0.0875	MIM	Mitochondrial inner membrane protein; role in mitophagy is disputed	0
		tif51A-3	0.740	0.0438			
	HYR1	*tif51A-1*	0.444	0.0685	IMS	Glutathione peroxidase; functions as hydroperoxide receptor	3
		tif51A-3	0.326	0.0391			
Others	PDR5	*tif51A-1*	2.151	0.0006		Plasma membrane ATP-binding cassette (ABC) transporter	3
		tif51A-3	3.851	0.0030			
	ECM16	*tif51A-1*	2.134	0.4299		Essential DEAH-box ATP-dependent RNA helicase specific to U3 snoRNP	9
		tif51A-3	2.696	0.0428			
	DBP2	*tif51A-1*	1.810	0.0731		ATP-dependent RNA helicase of the DEAD-box protein family	4
		tif51A-3	1.504	0.0237			
	SNQ2	*tif51A-1*	1.665	0.0403		Plasma membrane ATP-binding cassette (ABC) transporter	9
		tif51A-3	1.874	0.0070			
	MBF1	*tif51A-1*	1.559	0.1826		Transcriptional coactivator	0
		tif51A-3	1.493	0.0421			
	ABF2	*tif51A-1*	1.480	0.0308	MATRIX	Mitochondrial DNA-binding protein; involved in DNA replication and recombination	1
		tif51A-3	1.403	0.0514			
	RIB4	*tif51A-1*	1.335	0.0022	IMS	Lumazine synthase; catalyzes synthesis of immediate precursor to riboflavin	1
		tif51A-3	1.487	0.0004			
	IDP1	*tif51A-1*	1.280	0.0194	MATRIX	Mitochondrial NADP-specific isocitrate dehydrogenase	2
		tif51A-3	1.527	0.0058			
	HXK2	*tif51A-1*	1.268	0.0934	MS	Hexokinase isoenzyme 2; phosphorylates glucose in cytosol	2
		tif51A-3	1.540	0.0198			
	LSP1	*tif51A-1*	1.185	0.0277	MOM	Eisosome core component	5
		tif51A-3	0.837	0.0827			
	FMN1	*tif51A-1*	1.182	0.3869	MIM	Riboflavin kinase. produces riboflavin monophosphate (FMN)	1
		tif51A-3	0.634	0.0387			
	NAT1	*tif51A-1*	1.182	0.0310		Subunit of protein N-terminal acetyltransferase NatA	3
		tif51A-3	1.084	0.3228			
	HEM1	*tif51A-1*	1.146	0.4233	MATRIX	5-aminolevulinate synthase; catalyzes the first step in the heme biosynthetic pathway	5
		tif51A-3	0.595	0.0305			

	Gene	Strain	Relative 41/25 protein ratio vs WT [1]	p-value	Location [2]	Description [2]	Putative eIF5A motifs [3]
Others	FPR1	*tif51A-1*	1.024	0.9786		Peptidyl-prolyl cis-trans isomerase; acts as chaperone to prevent protein aggregation.	5
		tif51A-3	0.398	0.0012			
	PGK1	*tif51A-1*	1.107	0.4701		3-phosphoglycerate kinase; transfers phosphoryl groups to ADP to produce ATP	6
		tif51A-3	1.248	0.0119			
	PRE6	*tif51A-1*	0.927	0.2263	MS	Alpha 4 subunit of the 20S proteasome	4
		tif51A-3	0.791	0.0174			
	GPM1	*tif51A-1*	0.875	0.0540	IMS	Tetrameric phosphoglycerate mutase; participates in gluconegenesis	5
		tif51A-3	0.835	0.0253			
	NCE102	*tif51A-1*	0.866	0.2662		Protein involved in regulation of pheromone response and mating	0
		tif51A-3	0.646	0.0199			
	OYE2	*tif51A-1*	0.853	0.1319		Conserved NADPH oxidoreductase containing flavin mononucleotide (FMN)	3
		tif51A-3	0.754	0.0151			
	DUG1	*tif51A-1*	0.839	0.0057		Cys-Gly metallo-di-peptidase	6
		tif51A-3	0.799	0.0592			
	FBA1	*tif51A-1*	0.839	0.0181	MS	Fructose 1.6-bisphosphate aldolase; required for glycolysis and gluconeogenesis	2
		tif51A-3	0.984	0.6094			
	DNM1	*tif51A-1*	0.830	0.0329	MOM	Dynamin-related GTPase involved in mitochondrial organization	8
		tif51A-3	0.849	0.0464			
	YIM1	*tif51A-1*	0.811	0.2762		Aldehyde reductase; involved in detoxification of lignocellulose-derived aldehydes	5
		tif51A-3	0.474	0.0294			
	YDL086W	*tif51A-1*	0.810	0.1067		Putative carboxymethylenebutenolidase	0
		tif51A-3	0.729	0.0442			
	PIL1	*tif51A-1*	0.812	0.0610	MOM	Eisosome core component involved in endocytosis	3
		tif51A-3	0.694	0.0230			
	FRD1	*tif51A-1*	0.802	0.0436		Soluble fumarate reductase; may interact with ribosomes	3
		tif51A-3	0.898	0.2154			
	GNP1	*tif51A-1*	0.797	0.3180		Broad specificity amino acid permease; major serine permease	5
		tif51A-3	0.534	0.0442			
	LAT1	*tif51A-1*	0.795	0.0100	MATRIX	E2 component of the pyruvate dehydrogenase complex	0
		tif51A-3	0.683	0.0092			
	MSS116	*tif51A-1*	0.787	0.0269	MATRIX	Mitochondrial transcription elongation factor; DEAD-box protein	1
		tif51A-3	1.019	0.7656			
	ARG7	*tif51A-1*	0.785	0.0507	MATRIX	Mitochondrial ornithine acetyltransferase; catalyzes the fifth step in arginine biosynthesis	0
		tif51A-3	0.771	0.0406			
	SEC4	*tif51A-1*	0.775	0.0250	MOM	Rab family GTPase; essential for vesicle-mediated exocytic secretion and autophagy	0
		tif51A-3	0.694	0.0158			
	DPM1	*tif51A-1*	0.769	0.0576	MOM	Dolichol phosphate mannose synthase of ER membrane	0
		tif51A-3	0.759	0.0249			
	VPS21	*tif51A-1*	0.769	0.0279	MOM	Endosomal Rab family GTPase	0
		tif51A-3	0.669	0.0055			
	CYS4	*tif51A-1*	0.748	0.0885		Cystathionine beta-synthase; catalyzes the first committed step in cysteine biosynthesis	1
		tif51A-3	0.726	0.0253			
	NUP2	*tif51A-1*	0.747	0.0102		Nucleoporin involved in nucleocytoplasmic transport	5
		tif51A-3	0.678	0.0553			
	YKT6	*tif51A-1*	0.747	0.0455		Vesicle membrane protein (v-SNARE) with acyltransferase activity	0
		tif51A-3	0.739	0.0499			
	PMA1	*tif51A-1*	0.739	0.0202	MIM	Plasma membrane P2-type H+-ATPase; pumps prótons out of the cell	4
		tif51A-3	0.713	0.0167			
	ECM33	*tif51A-1*	0.738	0.0055		GPI-anchored protein involved in efficient glucose uptake	3
		tif51A-3	0.660	0.0165			
	RAD23	*tif51A-1*	0.718	0.0520		Proteasome-associated ubiquitin receptor; recruits substrates to the proteasome	4
		tif51A-3	0.541	0.0123			
	YCP4	*tif51A-1*	0.717	0.0360		Protein of unknown function; has sequence and structural similarity to flavodoxins	3
		tif51A-3	0.709	0.0290			
	CPR3	*tif51A-1*	0.719	0.2164		Mitochondrial peptidyl-prolyl cis-trans isomerasematr	0
		tif51A-3	0.504	0.0106			
	PRX1	*tif51A-1*	0.709	0.0189	MATRIX	Mitochondrial peroxiredoxin with thioredoxin peroxidase activity	2
		tif51A-3	0.572	0.0028			

	Gene	Strain	Relative 41/25 protein ratio vs WT [1]	p-value	Location [2]	Description [2]	Putative eIF5A motifs [3]
Others	VPS1	*tif51A-1*	0.680	0.0009	MOM	Dynamin-like GTPase required for vacuolar sorting	6
		tif51A-3	0.719	0.0077			
	ENO1	*tif51A-1*	0.672	0.0372		Enolase I. a phosphopyruvate hydratase	2
		tif51A-3	0.666	0.0293			
	YPT7	*tif51A-1*	0.669	0.0362		Enolase I. a phosphopyruvate hydratase	0
		tif51A-3	1.020	0.8286			
	AIM45	*tif51A-1*	0.667	0.0219		Putative ortholog of mammalian ETF-alpha; interacts with frataxin	4
		tif51A-3	0.908	0.7639			
	FAA1	*tif51A-1*	0.589	0.0120	MOM	Long chain fatty acyl-CoA synthetase; activates fatty acids	10
		tif51A-3	0.482	0.0046			
	RTN1	*tif51A-1*	0.579	0.0008		Reticulon protein; involved in nuclear pore assembly and tubular ER morphology	0
		tif51A-3	0.598	0.0007			
	LAP3	*tif51A-1*	0.573	0.0011		Cysteine aminopeptidase with homocysteine-thiolactonase activity	2
		tif51A-3	0.606	0.0108			
	DCS1	*tif51A-1*	0.568	0.0137		Non-essential hydrolase involved in mRNA decapping; activates Xrn1p	1
		tif51A-3	0.428	0.0057			
	YME1	*tif51A-1*	0.509	0.0266	MIM	Catalytic subunit of i-AAA protease complex; helps degradation of unfolded gene products.	7
		tif51A-3	0.682	0.1948			
	ADK1	*tif51A-1*	0.505	0.0859	IMS	Adenylate kinase. required for purine metabolism; controls ATP homeostasis	3
		tif51A-3	0.359	0.0281			
	DOP1	*tif51A-1*	0.502	0.0308		Protein involved in vesicular transport at trans-Golgi network	4
		tif51A-3	0.391	0.0120			
	SOD1	*tif51A-1*	0.502	0.1241	IMS	Cytosolic copper-zinc superoxide dismutase and sulfide oxidase	0
		tif51A-3	0.341	0.0373			
	EIS1	*tif51A-1*	0.486	0.0432		Component of the eisosome required for proper eisosome assembly	4
		tif51A-3	0.415	0.0453			
	CFT1	*tif51A-1*	0.478	0.0407		RNA-binding subunit of the mRNA cleavage and polyadenylation factor	8
		tif51A-3	0.706	0.2263			
	PST2	*tif51A-1*	0.470	0.0042		FMN-dependent NAD(P)H: quinone oxidoreductase	4
		tif51A-3	0.366	0.0011			
	ZEO1	*tif51A-1*	0.370	0.1195	MOM	Peripheral membrane protein of the plasma membrane; regulates cell integrity	0
		tif51A-3	0.294	0.0473			
	ALD4	*tif51A-1*	0.333	0.0015	MATRIX	Mitochondrial aldehyde dehydrogenase	3
		tif51A-3	0.182	0.0005			
	HXT7	*tif51A-1*	0.329	0.0266		FMN-dependent NAD(P)H:quinone oxidoreductase; induced by oxidative stress	2
		tif51A-3	0.281	0.0244			
	TDH1	*tif51A-1*	0.310	0.0002		Glyceraldehyde-3-phosphate dehydrogenase; involved in glycolysis and gluconeogenesis	2
		tif51A-3	0.377	4E-04			

[1] Relative 41°C/25°C protein ratio of the indicated eIF5A mutant strain respect to the wild-type.

[2] Information obtained from SGD (Saccharomyces Genome Database). MS, mitochondrial surface; MOM, mitochondrial outer membrane; MIM, mitochondrial inner membrane; IMS, mitochondrial intermembrane space.

[3] Number of eIF5A-dependent tripeptide motifs described in Pelechano and Alepuz, 2017.

* Down-regulated proteins in eIF5A mutants respect to wild-type but with a non-significant statistical value.

Table S2. Yeast strains used in this study

Name	Genotype	Source
**BY4741**	MATa *ura3Δ0 leu2Δ0 his3Δ1 met15Δ0*	Euroscarf
tif51A-1	BY4741 MATa *ura3Δ0 leu2Δ0 his3Δ1 met15Δ0 tif51A-1::kanR*	(Li *et al.,* 2011)
tif51A-3	BY4741 MATa *ura3Δ0 leu2Δ0 his3Δ1 met15Δ0 tif51A-3::kanR*	(Li *et al.,* 2011)
**PAY864**	BY4741 MATa *ura3Δ0 leu2Δ0 his3Δ1 met15Δ0 TIM50-3HA-his3MX6*	This study
**PAY866**	BY4741 MATa *ura3Δ0 leu2Δ0 his3Δ1 met15Δ0 tif51A-1::kanR TIM50-3HA-his3MX6*	This study
**PAY937**	BY4741 MATa *ura3Δ0 leu2Δ0 his3Δ1 met15Δ0 PDR5-GFP-his3MX6*	This study
**PAY938**	BY4741 MATa *ura3Δ0 leu2Δ0 his3Δ1 met15Δ0 tif51A-1::kanR PDR5-GFP-his3MX6*	This study
**PAY1066**	BY4741 MATa *ura3Δ0 leu2Δ0 his3Δ1 met15Δ0 TOM70-GFP-his3MX6*	This study
**PAY1067**	BY4741 MATa *ura3Δ0 leu2Δ0 his3Δ1 met15Δ0 tif51A-1::kanR TOM70-GFP-his3MX6*	This study
**PAY1068**	BY4741 MATa *ura3Δ0 leu2Δ0 his3Δ1 met15Δ0 CYC1-GFP-his3MX6*	This study
**PAY1069**	BY4741 MATa *ura3Δ0 leu2Δ0 his3Δ1 met15Δ0 tif51A-1::kanR CYC1-GFP-his3MX6*	This study
**PAY1072**	BY4741 MATa *ura3Δ0 leu2Δ0 his3Δ1 met15Δ0 ILV2-GFP-his3MX6*	This study
**PAY1073**	BY4741 MATa *ura3Δ0 leu2Δ0 his3Δ1 met15Δ0 tif51A-1::kanR ILV2-GFP-his3MX6*	This study
**PAY1078**	BY4741 MATa *ura3Δ0 leu2Δ0 his3Δ1 met15Δ0 TIM50-GFP-his3MX6*	This study
**PAY1079**	BY4741 MATa *ura3Δ0 leu2Δ0 his3Δ1 met15Δ0 tif51A-1::kanR TIM50-GFP-his3MX6*	This study
**PAY1080**	BY4741 MATa *ura3Δ0 leu2Δ0 his3Δ1 met15Δ0 YTA12-GFP-his3MX6*	This study
**PAY1081**	BY4741 MATa *ura3Δ0 leu2Δ0 his3Δ1 met15Δ0 tif51A-1::kanR YTA12-GFP-his3MX6*	This study
**PAY1085**	BY4741 MATa *ura3Δ0 leu2Δ0 his3Δ1 met15Δ0 TIM50Δ7Pro-GFP-his3MX6*	This study
**PAY1086**	BY4741 MATa *ura3Δ0 leu2Δ0 his3Δ1 met15Δ0 tif51A-1::kanR TIM50Δ7Pro-GFP-his3MX6*	This study
**PAY1107**	BY4741 MATa *ura3Δ0 leu2Δ0 his3Δ1 met15Δ0 TIM50-GFP-his3MX6 HSP104-RFP-clonNAT*	This study
**PAY1113**	BY4741 MATa *ura3Δ0 leu2Δ0 his3Δ1 met15Δ0 tif51A-1::kanR TIM50-GFP-his3MX6 HSP104-RFP-clonNAT*	This study
**PAY1142**	BY4741 MATa *ura3Δ0 leu2Δ0 his3Δ1 met15Δ0 CYC1-GFP-his3MX6 HSP104-RFP-clonNAT*	This study
**PAY1144**	BY4741 MATa *ura3Δ0 leu2Δ0 his3Δ1 met15Δ0 tif51A-1::kanR CYC1-GFP-his3MX6 HSP104-RFP-clonNAT*	This study

Table S3. Plasmids used in this study

Name	Plasmid description	Source
**PA201**	pFA6a-3HA-HIS3MX6	(Longtine *et al.,* 1998)
**PA242**	pFA6a-GFP-HIS3MX6	(Longtine *et al.,* 1998)
**PA354**	pMK46-IAA17-kanMX	Dr. Ethel Queralt
**PA367**	pYM43-Redstar2-clonNAT	Euroscarf
**PA386**	pYES2-pGAL-FLAG-htt25QP-GFP-URA3	(Berglund *et al.*, 2017)
**PA388**	pYES2-pGAL-FLAG-TIM50-GFP-URA3	This study
**TTP76**	pRS406-GPDp-Su9-mCherry-URA3	Dr. Brian M. Zid
**TTP80**	pRS405-CYC1p-MS2-4xGFP-LEU2	Dr. Brian M. Zid
**TTP145**	pRS403 TIM50p-TIM50mts(1-300)-TIM50orf-flagiRFP-TIM50ter-MS2tag	Dr. Brian M. Zid
**ZP447**	pAG306-ptetO$_7$-TIM505 'UTR-TIM50-nLuc-CYC1term-URA3	Dr. Brian M. Zid
**ZP448**	pAG306-ptetO$_7$-TIM505 'UTR-TIM50Δ7Pro-nLuc-CYC1term-URA3	Dr. Brian M. Zid
**ZP562**	pAG306-ptetO$_7$-TIM505 'UTR-SDH2-nLuc-CYC1term-URA3	Dr. Brian M. Zid
**ZP603**	pAG306-ptetO$_7$-TIM505 'UTR-CYC1-nLuc-CYC1term-URA3	This study
**ZP605**	pAG306-ptetO$_7$-TIM505 'UTR-COX5A-nLuc-CYC1term-URA3	This study

Table S4. Oligonucleotides used in this study

Primer	Sequence (5'-3')
**Gene expression detection by RT-qPCR**	
ACT1-F	TCGTTCCAATTTACGCTGGTT
ACT1-R	CGGCCAAATCGATTCTCAA
ATP1-F	AGACCTGCCATTAACGTTGG
ATP1-R	AGCAAAAGCAGCGACTTCTC
CIS1-F	TGCAGAGTGGGTAGCATGTC
CIS1-R	TGGGCAGCCTTGAGTAAATC
COX5A-F	ATCCAGATGGGAGAACATGC
COX5A-R	AGCTTGCTTTTCAGGCTCAG
CYC1-F	AGATGTCTACAATGCCACACC
CYC1-R	CCCTTCAGCTTGACCAGAGT
EFT2-F	TGTTCATCAAGGCCATTCAA
EFT2-R	GTTACCGGCTGGACAGTCAT
GFP-F	CACATGAAGCAGCACGACTT
GFP-R	GGTCTTGTAGTTGCCGTCGT
GRE2-F	GCCTTCCAAAAGAGGGAAAC
GRE2-R	ATGGGTAGCACCAGAACCTG
HSP60-F	CACTGATCCAAAGTCGAGCA
HSP60-R	CAAGAGCTTCACCGTCAACA
MSP1-F	ACGGCAACCTTAAAAGCTGA
MSP1-R	CCTCCTCAAAAAACGCATCAT
NLUC-F	AGAACAAGGTGGTGTTTCTTCT
NLUC-R	CCCATTTGATCACCAGATAAACCT
PDR1-F	TCCAAATGCGAGATTTTTCC
PDR1-R	CGAAGATGGGGTTGAAGGTA
PDR3-F	AGATGGGATTGTCTCGTTGG
PDR3-R	CTGAAATCCTTCGGCAAGAG
PDR5-F	GTACCGTGGTTGGAGCTGTT
PDR5-R	GAAACCACGCCATTTGTCTT
PDR15-F	CCAAGGTCGGAAACGATCTA
PDR15-R	CAATGTCAGCCTGGGTTTTT
PET9-F	AGGCCATGTTTGGTTTCAAG
PET9-R	TCAAACCGTTGAATTGACGA
POR1-F	AAACCGGCTTGGGTCTAACT
POR1-R	GACGCCTGGAGTCAAAGAAG
RPP2B-F	GGAAGGTAAGGGCTCTTTGG
RPP2B-R	TTCTTCAGCAGCATCACCAC
TIM50-F	TCTGCGTTGACAGGTACTGC
TIM50-R	AATCAGGGAAAGGTGGCTCT

TOM20-F	CCGCAATTCAGGAAAGTGTT
TOM20-R	CCTTTTGCAGCTTCACTTCC
TOM70-F	GACCCAAGAAGTGAGCAAGC
TOM70-R	AGCGGCTTCAGCAAAAGTAA
**Gene tagging by PCR**	
CYC1-F2	AAAGACAGAAACGACTTAATTACCTACTTGAAAAAAGCCTGTGAGCGGATCCCCGGGTTAATTAA
CYC1-R1	TGACATAACTAATTACATGATATCGACAAAGGAAAAGGGGCCTGTGAATTCGAGCTCGTTTAAAC
HSP104-RFP-F	GATGACGATAATGAGGACAGTATGGAAATTGATGATGACCTAGATCGTACGCTGCAGGTCGAC
HSP104-RFP-R	TACTGCTTCTTGTTCGAAAGTTTTTTAAAAATCACACTATATTAAATCAATCGATGAATTCGAGCTCG
ILV2-F2	AGACAACAGACTGAATTACGTCATAAGCGTACAGGCGGTAAGCACCGGATCCCCGGGTTAATTAA
ILV2-R1	TGCATTTTTTACTGAAAATGCTTTTGAAATAAATGTTTTTGAAATGAATTCGAGCTCGTTTAAAC
PDR5-F2	TGGTTAGCAAGAGTGCCTAAAAAGAACGGTAAACTCTCCAAGAAACGGATCCCCGGGTTAATTAA
PDR5-R1	GTCCATCTTGGTAAGTTTCTTTTCTTAACCAAATTCAAAATTCTAGAATTCGAGCTCGTTTAAAC
TIM50-F2	TTATTTGAAGAGGAAAAGAAAAAAGAAGAAGATTGCTGAATCCAAACGGATCCCCGGGTTAATTAA
TIM50-R1	CACACATAGATACGTAGATACATGAGAAGAGGGTTTACATGAAAAGAATTCGAGCTCGTTTAAAC
TOM70-F2	AAGATTCAAGAAACTTTAGCTAAATTACGCGAACAGGGTTTAATGCGGATCCCCGGGTTAATTAA
TOM70-R1	TAGTTTTTGTCTTCTCCTAAAAGTTTTTAAGTTTATGTTTACTGTGAATTCGAGCTCGTTTAAAC
YTA12-F2	GAAGAAAAAAACGAAAAACGTAATGAGCCTAAGCCATCTACAAACCGGATCCCCGGGTTAATTAA
YTA12-R1	ATATGTAGAACAGTCTTCCTCCATTTCTTTGTATTGTGAAATATCGAATTCGAGCTCGTTTAAAC
**Proline deletion by PCR**	
TIM50-delPro-F	CCTACTTCCAAGAGCCACCTTTCCCTGATTTACTACCAAAGGCCATTAACTCTTG
TIM50-delPro-R	CACACATAGATACGTAGATACATGAGAAGAGGGTTTACATGAAAA
**Cloning into pYES2 plasmid**	
TIM50-F3	AAGCTTGGTACCGCCATGGACTACAAGGACGACGATGACAAGCTGCTGTCCATTTTAAGAAATTC
TIM50-R3	CACCCCGGTGAACAGCTCCTCGCCCTTGCTCACCAGGGATCCCCCTTTGGATTCAGCAATCTTCT
**Cloning into ZP446 plasmid**	
ZP446-F	ATGGTTTTTACTTTAGAAGATTTTG
ZP446-R	TGCAAGCGGGTGATTTTTGGAAGTTTATTCTAGC
CYC1-F	CCAAAAATCACCCGCTTGCAATGACTGAATTCAAGGCCGGTTCTGCTAAG
CYC1-R	CAACAAAATCTTCTAAAGTAAAAACCATCTCACAGGCTTTTTTCAAGTAGGTAAT
COX5A-F	CCAAAAATCACCCGCTTGCAATGTTACGTAACACTTTTACTAGAGCTGGT
COX5A-R	CAACAAAATCTTCTAAAGTAAAAACCATTTTAGATTGGACCTGAGAATAACCAC
